# Cervical arthroplasty versus anterior cervical discectomy in the treatment of symptomatic cervical spondylosis: A protocol

**DOI:** 10.1097/MD.0000000000022145

**Published:** 2020-09-11

**Authors:** Yi Tong, Xufeng Jia, Yunlong Zhou, Daxiong Feng, Dechao Yuan

**Affiliations:** a Department of Orthopedics, Zigong No.4 People's Hospital; b Department of Orthopedics, The People's Hospital of Jian Yang; c Department of Orthopedics, The People's Hospital of Le Shan; d Department of Orthopedics, The Affiliated Hospital of Southwest Medical University, Sichuan, PR China.

**Keywords:** cervical spondylosis, anterior cervical discectomy and fusion, cervical disc arthroplasty, study protocol

## Abstract

**Background:**

Anterior cervical discectomy and fusion (ACDF) and cervical disc arthroplasty (CDA) are both the effective techniques in treatment of cervical spondylosis. The purpose of this present retrospective cohort research was to assess the efficacy and safety of ACDF and CDA in treating the symptomatic cervical spondylosis over the 6-year follow-up.

**Methods:**

From our registry database, we identified retrospectively patients who received CDA or ACDF in our academic institutions from 2012 to 2015. The study was approved by the Institutional Review Board in Zigong No.4 People's Hospital (Z10058072). All the subjects who participated in this trial were informed consent in writing. The inclusion criteria were the degenerative disc diseases between C3-7 resulting in myelopathy or radiculopathy, which was unresponsive to the conservative treatment. The clinical results were determined via Short Form-36, and neck disability index, numerical scoring scales for complications, arm pain and neck pain. The radiographic assessment contained the cervical lordosis, and the motion range of the functional spinal unit and total cervical spine. The routine follow-up was performed to collect the data of radiographic and clinical assessment at 6, 12, 24, 48, and 72 months before and after the surgery.

**Results:**

This study had limited inclusion and exclusion criteria and a well-controlled intervention. It was assumed that both techniques could obtain the similar postoperative effects.

**Trial registration:**

This study protocol was registered in Research Registry (researchregistry5878).

## 1 Introduction

Cervical spondylosis is a kind of chronic degenerative disease of cervical spine. It affects the intervertebral disks and vertebral bodies in the neck, leading to ligament hypertrophy, osteophytes, and herniated intervertebral disks.^[1,2]^ This may ultimately lead to spinal cord and nerve roots compression. Tingling, weakness, and numbness, headache and neck stiffness, as well as arm or neck pain are the common symptoms of cervical spondylosis. Numbness, pain, and other symptoms are reported to be associated with insomnia and depression.^[3–5]^

Anterior cervical discectomy and fusion (ACDF) has yielded very significant results in the majority of patients with persistent nerve roots who have not responded to the nonsurgical approaches. For decades, ACDF has been extensively utilized because of its high clinical success rate and it is regarded as a gold standard for the symptomatic cervical spondylosis surgical treatment.^[6–8]^ Nevertheless, there are currently a number of unique adverse events related to this procedure, containing adjacent-level disc disease.^[9]^ Cervical disc arthroplasty (CDA) is also an effective method to treat the degenerative cervical disc disease. The purpose of CDA is to avoid the fusion side effects, at the same time, maintain segmental motion and normal disc height.^[10,11]^ It also avoids the complications due to cervical immobilization and anterior cervical plating.^[12]^ Nevertheless, over time, some studies have shown that such surgery can produce certain adverse reactions, including the heterotopic ossification and increased arm pain or neck pain after operation.^[11,13]^

A lot of studies have compared the efficacy of ACDF and CDA in treating the symptomatic cervical spondylosis.^[7–11]^ As far as we know, there are few investigations comparing postoperative results of patients with 2 level continuous cervical spondylosis, namely the parents received CDA with Prestige-LP prosthesis and the patients received ACDF with Zero-P device. The purpose of this present retrospective cohort research was to assess the efficacy and safety of ACDF and CDA in treating the symptomatic cervical spondylosis over the 6-year follow-up. It was assumed that both techniques could obtain the similar postoperative effects.

## 2 Materials and methods

### 2.1 Study design

From our registry database, we identified retrospectively patients who received CDA or ACDF in our academic institutions from 2012 to 2015. The study was approved by the Institutional Review Board in Zigong No.4 People's Hospital (Z10058072). All the subjects who participated in this trial were informed consent in writing. We also registered this study with the research registry (researchregistry5878). This current study was conducted and reported on the basis of the requirements of Strengthening the Reporting of Observational studies in the Epidemiology checklist.

### 2.2 Inclusion and exclusion criteria

The inclusion criteria were the degenerative disc diseases between C3-7 resulting in myelopathy or radiculopathy, which was unresponsive to the conservative treatment. The exclusion criteria of the cervical arthroplasty included history of cervical spine surgery, the posterior longitudinal ligament ossification, and active infection, simple cervical axial pain, as well as the acute spinal injury, or the radiographic signs of instability.

### 2.3 Surgical techniques

All the operations were conducted under the condition of general anesthesia in laminar flow operating room and conducted via a same senior surgeon. The patient underwent the standard Smith-Robinson anterior approach that exposed proper levels of symptomatic cervical spine, followed by a proper neurological structures decompression and complete discectomy. In group ACDF, the patients were fused with an anterior titanium structure (Zero-P device) and an allograft structure. And in group CDA, in accordance with intraoperative fluoroscopy guidance, the Prestige LP (Medtronic, Memphis, TN) artificial disc was implanted in the patients in this series. Afterward, the closed drainage catheter was placed, and wound of each patient was sealed layer by layer.

### 2.4 Outcomes assessment

The clinical results were determined via Short Form-36, and neck disability index, numerical scoring scales for complications, arm pain and neck pain. The data were collected through 2 specialist nurse assistants during the outpatient period under the supervision of physicians. The radiographic assessment contained the cervical lordosis, and the motion range of the functional spinal unit and total cervical spine. The routine follow-up was performed to collect the data of radiographic and clinical assessment at 6, 12, 24, 48, and 72 months before and after the surgery (Tables 1 and 2).

**Table 1:**
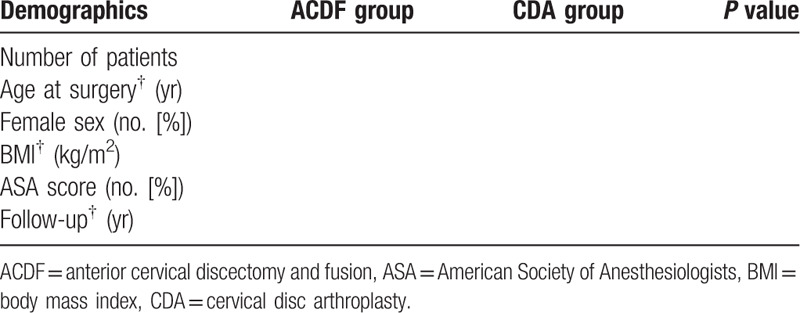
Patient baseline demographics.

**Table 2:**
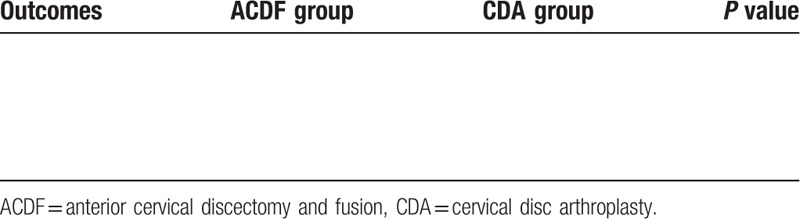
Clinical and radiographic outcomes.

### 2.5 Statistical analysis

SPSS 20.0 software was utilized for statistical calculation. For all the comparisons, a 0.05 alpha level was selected to indicate significance. Wilcoxon test or *t* test were utilized for the means comparisons between these groups, according to the distribution of data. And chi-square test was utilized for the comparisons of proportion. At the same time, the mixed-model analysis of variance was utilized to study the effect of both rehabilitation and time on the specific results. Post hoc tests utilized the correction of Tukey-Kramer to adjust multiple comparisons.

## 3 Discussion

The patients with cervical radiculopathy can be successfully treated with non-surgical treatment strategies, such as physical therapy, anti-inflammatory and analgesic drugs and axial traction. The most familiar indications of surgery for the degenerative cervical spondylosis are intractable radiculopathy and progressive neurological dysfunction, which are difficult to accept with appropriate non-surgical treatment.^[14]^ Both CDA and ACDF are effective approaches to treat the symptomatic cervical spondylosis. The purpose of this present retrospective cohort research was to assess the efficacy and safety of ACDF and CDA in treating the symptomatic cervical spondylosis over the 6-year follow-up. It was assumed that both techniques could obtain the similar postoperative effects.

Limitations of our current work include limitations inherent in any retrospective cohort research, including the observational bias and selection possibility. Long-term follow-up (between 10 and 15 years) was also not involved in this study, as our current work relied on the electronic medical records preserved since 2012. The authors realize that long-term follow-up is essential to determine the difference in complications between the 2 methods.

## Author contributions

**Conceptualization**: Yi Tong.

**Data curation**: Yi Tong, Xufeng Jia, Dechao Yuan.

**Formal analysis**: Yi Tong, Xufeng Jia.

**Funding acquisition**: Yi Tong.

**Investigation**: Yi Tong, Xufeng Jia.

**Methodology**: Yunlong Zhou.

**Resources**: Daxiong Feng.

**Software**: Daxiong Feng.

**Supervision**: Yi Tong.

**Validation**: Dechao Yuan.

**Visualization**: Daxiong Feng.

**Writing – original draft**: Yi Tong, Xufeng Jia.

**Writing – review & editing**: Yunlong Zhou.

## References

[1] Stoffman MR Roberts MS King JJ Cervical spondylotic myelopathy, depression, and anxiety: a cohort analysis of 89 patients. Neurosurgery 2005;57:307 13. 16094160 10.1227/01.neu.0000166664.19662.43

[2] Wang C Tian F Zhou Y The incidence of cervical spondylosis decreases with aging in the elderly, and increases with aging in the young and adult population: a hospital-based clinical analysis. Clin Interv Aging 2016;11:47 53. 26834465 10.2147/CIA.S93118 PMC4716725

[3] Paanalahti K Holm LW Magnusson C The sex-specific interrelationship between spinal pain and psychological distress across time in the general population. Spine J 2014;14:1928 35. 24262854 10.1016/j.spinee.2013.11.017

[4] Hartvigsen J Christensen K Frederiksen H Back and neck pain exhibit many common features in old age: a population-based study of 4,486 Danish twins 70-102 years of age. Spine (Phila Pa 1976) 2004;29:576 80. 15129076 10.1097/01.brs.0000099394.18994.2f

[5] Lv Y Tian W Chen D The prevalence and associated factors of symptomatic cervical Spondylosis in Chinese adults: a community-based cross-sectional study. BMC Musculoskelet Disord 2018;19:325. 30205836 10.1186/s12891-018-2234-0 PMC6134586

[6] Segebarth B Datta JC Darden B Incidence of dysphagia comparing cervical arthroplasty and ACDF. SAS J 2010;4:3 8. 25802643 10.1016/j.esas.2009.12.001 PMC4365608

[7] Lu H Peng L Efficacy and safety of Mobi-C cervical artificial disc versus anterior discectomy and fusion in patients with symptomatic degenerative disc disease: a meta-analysis. Medicine (Baltimore) 2017;96:e8504. 29245217 10.1097/MD.0000000000008504 PMC5728832

[8] Li Y Shen H Khan KZ Comparison of multilevel cervical disc replacement and multilevel anterior discectomy and fusion: a systematic review of biomechanical and clinical evidence. World Neurosurg 2018;116:94 104. 29753897 10.1016/j.wneu.2018.05.012

[9] Anderson PA Subach BR Riew KD Predictors of outcome after anterior cervical discectomy and fusion: a multivariate analysis. Spine (Phila Pa 1976) 2009;34:161 6. 19139666 10.1097/BRS.0b013e31819286ea

[10] Anderson PA Sasso RC Hipp J Kinematics of the cervical adjacent segments after disc arthroplasty compared with anterior discectomy and fusion: a systematic review and meta-analysis. Spine (Phila Pa 1976) 2012;37:S85 95. 22885834 10.1097/BRS.0b013e31826d6628

[11] Aragonés M Hevia E Barrios C Polyurethane on titanium unconstrained disc arthroplasty versus anterior discectomy and fusion for the treatment of cervical disc disease: a review of level I-II randomized clinical trials including clinical outcomes. Eur Spine J 2015;24:2735 45. 26363559 10.1007/s00586-015-4228-z

[12] Zhao GS Zhang Q Quan ZX Mid-term efficacy and safety of cervical disc arthroplasty versus fusion in cervical spondylosis: a systematic review and meta-analysis. Biomed Rep 2017;6:159 66. 28357067 10.3892/br.2016.823 PMC5351268

[13] Malham GM Parker RM Ellis NJ Cervical artificial disc replacement with ProDisc-C: clinical and radiographic outcomes with long-term follow-up. J Clin Neurosci 2014;21:949 53. 24417795 10.1016/j.jocn.2013.09.013

[14] Mummaneni PV Burkus JK Haid RW Clinical and radiographic analysis of cervical disc arthroplasty compared with allograft fusion: a randomized controlled clinical trial. J Neurosurg Spine 2007;6:198 209. 17355018 10.3171/spi.2007.6.3.198

